# Metagenomes and Metagenome-Assembled Genomes from Microbial Communities Fermenting Ultrafiltered Milk Permeate

**DOI:** 10.1128/mra.00293-22

**Published:** 2022-06-30

**Authors:** Kevin A. Walters, Kevin S. Myers, Hui Wang, Nathaniel W. Fortney, Abel T. Ingle, Matthew J. Scarborough, Timothy J. Donohue, Daniel R. Noguera

**Affiliations:** a Great Lakes Bioenergy Research Center, University of Wisconsin-Madison, Madison, Wisconsin, USA; b Wisconsin Energy Institute, University of Wisconsin-Madison, Madison, Wisconsin, USA; c Department of Bacteriology, University of Wisconsin-Madison, Madison, Wisconsin, USA; d Department of Civil and Environmental Engineering, University of Wisconsin-Madison, Madison, Wisconsin, USA; e Department of Civil and Environmental Engineering, University of Vermont, Burlington, Vermont, USA; Indiana University, Bloomington

## Abstract

Fermentative microbial communities can be utilized for the conversion of various agroindustrial residues into valuable chemicals. Here, we report 34 metagenomes from anaerobic bioreactors fed lactose-rich ultrafiltered milk permeate and 278 metagenome-assembled genomes (MAGs). These MAGs can inform future studies aimed at generating renewable chemicals from dairy and other agroindustrial residues.

## ANNOUNCEMENT

**FIG 1:**
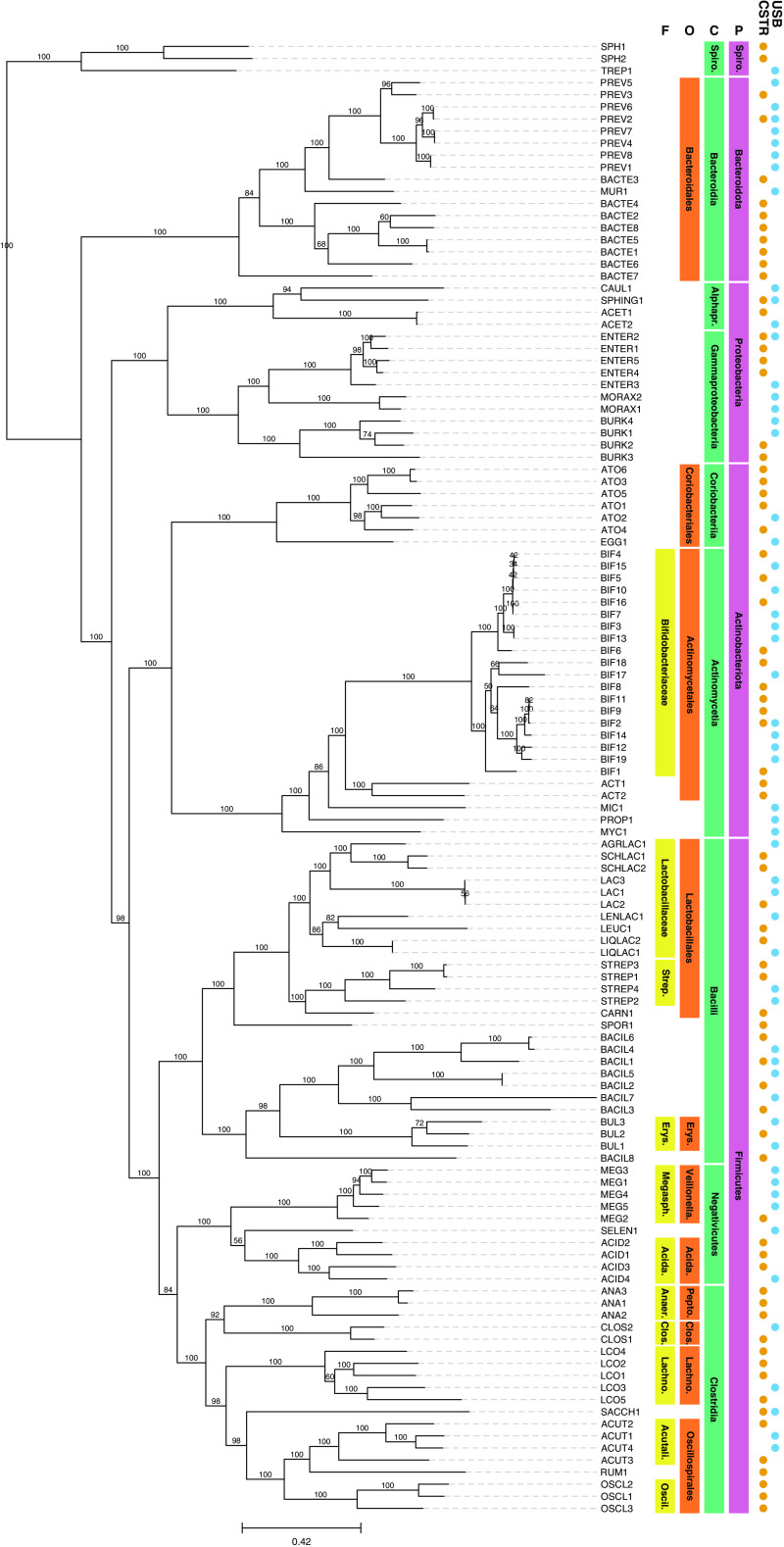
Phylogenic tree of dRep representative bacterial MAGs and their presence in two bioreactors (CSTR and USB) fermenting ultrafiltered milk permeate. ACET, *Acetobacter*; ACID, *Acidaminococcaceae*; ACT, *Actinomycetaceae*; ACUT, *Acutalibacteraceae*; AGRLAC, *Agrilactobacillus*; ANA, *Anaerovoracaceae*; ATO, *Atopobiaceae*; BACIL, *Bacilli*; BACTE, *Bacteroidales*; BIF, *Bifidobacterium*; BUL, *Bulleidia*; BURK, *Burkholderiaceae*; CARN, *Carnobacteriaceae*; CAUL, *Caulobacteraceae*; CLOS, *Clostridium*; EGG, *Eggerthellaceae*; ENTER, Enterobacteriaceae; LAC, *Lactobacillus*; LCO, *Lachnospiraceae*; LENLAC, *Lentilactobacillus*; LEUC, *Lecuonostoc*; LIQLAC, *Liquorilactobacillus*; MEG, *Megasphaera*; MIC, *Microbacteriaceae*; MORAX, *Moraxellaceae*; MUR, *Muribaculaceae*; MYC, *Mycobacteriaceae*; OSCL, *Oscillospiraceae*; PREV, *Prevotella*; PROP, *Propionibacteriaceae*; RUM, *Ruminococcaceae*; SACCH, *Saccharofermentans*; SCHLAC, *Schleiferilactobacillus*; SELEN, *Selenomonadaceae*; SPH, *Sphaerochaetaceae*; SPHING, *Sphingobium*; SPOR, *Sporolactobacillaceae*; STREP, *Streptococcaceae*; TREP, Treponema. Higher taxonomic levels are labeled, from left to right, family (F), order (O), class (C), and phylum (P). *Spiro.*, *Spirochaetota*; *Alphapr.*, *Alphaproteobacteria*; *Spiro.*, *Spirochaetia*; *Acida.*, *Acidaminococcales*; *Clos*., *Clostridiales*; *Erys.*, *Erysipelotrichales*; *Lachno*., *Lachnospirales*; *Pepto*., *Peptostreptococcales*; *Veillonella*., *Veillonellales*; *Acida.*, *Acidaminococcaceae*; *Acutali*., *Acutalibacteraceae*; *Anaer*., *Anaerovoracaceae*; *Clos*., *Clostridiaceae*; *Erys.*, *Erysipelotrichaceae*; *Lachno*, *Lachnospiraceae*; *Megasph*., *Megasphaeraceae*; *Oscil*., *Oscillospiraceae*; *Strep.*, *Streptococcaceae*. The phylogenetic tree was generated in RAxML-ng (14) with 500 bootstraps using the concatenation of 120 bacterial single-copy marker genes (Bac120) identified by GTDB-Tk (13). Bootstrap values greater than 50 are shown. The scale bar represents evolutionary distance and indicates the number of nucleotide substitutions per sequence site.

The metagenomes reported here originate from two anaerobic bioreactors, i.e., a continuously stirred tank reactor (CSTR) and an upflow sludge blanket reactor (USB), operated to investigate the valorization of agroindustrial residues via fermentation. Both bioreactors were inoculated with acid-phase anaerobic digester sludge from the Nine Springs Wastewater Treatment Plant (Madison, WI, USA) and fed ultrafiltered milk permeate amended with ammonium chloride as a nitrogen source. The CSTR was operated at pH 5.5 and 35°C, whereas the USB was operated at pH 5.5 and 21°C. DNA was periodically extracted from the bioreactors using a phenol-chloroform extraction procedure described by Scarborough et al. (1) but omitting the bead-beating step. DNA aliquots of 500 ng (27 samples) and 3,000 ng (7 samples) were submitted to the Joint Genome Institute (JGI) (Berkeley, CA, USA) for paired-end 2 × 150-bp NovaSeq S4 sequencing (Illumina, Inc., San Diego, CA, USA) and single-molecule real-time (SMRT), long-read sequencing using a Sequel II platform (Pacific Biosciences, Inc. [PacBio], Menlo Park, CA, USA), respectively. Illumina libraries were end repaired, A tailed, and ligated with Illumina adapters using the KAPA HyperPrep kit (Roche, USA) as described (2). PacBio library construction included shearing of genomic DNA to 6 to 10 kb (size selection with BluePippin; Sage Science, USA) and ligation using the SMRTbell Express template preparation v2.0 kit following the standard protocol (PacBio). All software used default parameters unless otherwise noted. Illumina reads were filtered and error corrected using bbcms (v38.86) (mincount=2, highcountfraction=0.6) (3), assembled with metaSPAdes (v3.14.1) (4), and mapped with BBMap (v38.86) (ambiguous=random) (3) following the JGI Metagenome Workflow (2). PacBio reads were filtered using BBtools (v38.87/38.88, rqc.filter2.sh) (3), and CCS reads were assembled using metaFlye (v2.8.1-b1676) (5), polished with subreads using GCpp (v1.0.0-SL-release-8.0.0) (https://github.com/PacificBiosciences/gcpp), and mapped using minimap2 (v2.17-r941) (6). For all libraries, contigs were binned with MetaBAT (v2:2.15) (7). The resulting Illumina libraries contained between 71 and 126 million 150-bp reads, and the PacBio libraries contained between 21,000 and 1,122,000 reads, 5 to 8 kb in average length. The resulting metagenome-assembled genomes (MAGs) were annotated using the JGI Metagenome Annotation Pipeline (MAP) (v5.0.23) (8) and the NCBI Prokaryotic Genome Annotation Pipeline (PGAP) (v6.0) (9). To improve MAG quality, contaminant contigs from all MAGs were identified and removed using ProDeGe (v2.3) (10) and custom tetranucleotide frequency analysis scripts (run.GC.sh and Calculating_TF_Correlations.R [https://github.com/GLBRC/metagenome_analysis]). All refined MAGs were dereplicated using dRep (v3.2.2) (dereplicate command with –conW 0.5 and –N50W 5 custom parameters) (11) by clustering MAGs by identity and selecting the highest-quality MAG as a representative for each cluster (Table 1). Quality statistics were obtained using CheckM (v1.0.11) (12), and MAGs with over 75% completeness were retained for further analysis. Taxonomy was assigned for dRep representative MAGs using GTDB-Tk (v1.5.1, database release 202) (13). RAxML-NG (v0.9.0) (14) and TreeViewer (v2.0.1) were used to generate and visualize a phylogenetic tree containing dRep representative MAGs (Fig. 1). We report 278 annotated MAGs from 34 samples, grouped into 123 dereplicated clusters that describe the microbial community composition of the two bioreactors (Table 1). These data contribute to the knowledgebase of microbial communities bioconverting agroindustrial residues (1, 15–25).

**TABLE 1:** MAG statistics and genome accession numbers

		Sample origin^*c*^																			
Strain name^*a*^	Code^*b*^	Reactor	Day	ANIm^*d*^	dRep^*e*^	GTDB-Tk classification	Reference genome^*f*^	Sequencing platform	Completeness (%)	Contamination (%)	MAG size (Mbp)	No. of contigs	*N*_50_ (Mbp)	GC content (%)	No. of tRNAs	No. of 5S rRNAs	No. of 16S rRNAs	No. of 23S rRNAs	GenBank accession no.^*g*^	SRA accession no.^*h*^	No. of raw reads aligned to MAG (×1,000)^*i*^
UW_MP_ACET1_1	ACET1	CSTR	120		122	d__Bacteria;p__Proteobacteria;c__Alphaproteobacteria;o__Acetobacterales;f__Acetobacteraceae;g__Acetobacter;s__Acetobacter lovaniensis	GCF_014207635.1	Illumina NovaSeq S4	96.02	0	2.163	15	0.222	58.9	38	0	0	0	JALCMR000000000	SRX12655888	348
UW_MP_ACET1_2		CSTR	126	0.9999	107			Illumina NovaSeq S4	85.22	0.5	2.257	92	0.0302	58.5	34	0	0	0	JALCMZ000000000	SRX12655887	176
UW_MP_ACET2_1	ACET2	USB	148		121	d__Bacteria;p__Proteobacteria;c__Alphaproteobacteria;o__Acetobacterales;f__Acetobacteraceae;g__Acetobacter;s__Acetobacter fabarum	GCF_011516925.1	Illumina NovaSeq S4	95.52	0.25	2.282	10	0.2426	59.0	38	0	0	0	JALCDC000000000	SRX12657082	496
UW_MP_ACID1_1	ACID1	CSTR	44		132	d__Bacteria;p__Firmicutes_C;c__Negativicutes;o__Acidaminococcales;f__Acidaminococcaceae;g__Acidaminococcus;s__	NA	PacBio Sequel II	100	0.6	3.057	1	3.057	48.1	52	3	3	3	JAKVOM000000000	SAMN18243037	8
UW_MP_ACID1_2		CSTR	54	0.9999	118			Illumina NovaSeq S4	95.21	0	2.532	74	0.0442	48.2	50	1	0	0	JALCTG000000000	SRX12654502	922
UW_MP_ACID1_3		CSTR	49	1.0000	112			Illumina NovaSeq S4	88.62	0	2.301	63	0.046	48.5	47	1	0	0	JALCSY000000000	SRX12654484	3,262
UW_MP_ACID1_4		CSTR	36	1.0000	108			Illumina NovaSeq S4	84.43	0	2.098	57	0.0458	48.4	47	1	0	0	JALCSK000000000	SRX12654483	8,659
UW_MP_ACID2_1	ACID2	CSTR	24		122	d__Bacteria;p__Firmicutes_C;c__Negativicutes;o__Acidaminococcales;f__Acidaminococcaceae;g__;s__	NA	PacBio Sequel II	95.76	6.59	2.327	18	0.1935	40.6	50	1	1	1	JAKVOW000000000	SAMN18243588	3
UW_MP_ACID2_2		CSTR	36	0.9903	99			Illumina NovaSeq S4	77.64	0	1.455	95	0.0161	40.8	34	0	0	0	JALCSJ000000000	SRX12654483	132
UW_MP_ACID3_1	ACID3	CSTR	36		118	d__Bacteria;p__Firmicutes_C;c__Negativicutes;o__Acidaminococcales;f__Acidaminococcaceae;g__Succiniclasticum;s__Succiniclasticum sp900314855	GCA_900314855.1	Illumina NovaSeq S4	95.09	0	1.769	31	0.0707	59.7	45	1	2	1	JALCSI000000000	SRX12654483	174
UW_MP_ACID4_1	ACID4	USB	0		101	d__Bacteria;p__Firmicutes_C;c__Negativicutes;o__Acidaminococcales;f__Acidaminococcaceae;g__;s__	NA	Illumina NovaSeq S4	79.92	1.2	1.721	58	0.0383	51.9	25	4	1	0	JAKVKY000000000	SRX12655975	168
UW_MP_ACT1_1	ACT1	CSTR	78		127	d__Bacteria;p__Actinobacteriota;c__Actinomycetia;o__Actinomycetales;f__Actinomycetaceae;g__Ancrocorticia;s__	NA	Illumina NovaSeq S4	99.58	0.84	2.228	16	0.2166	58.1	45	0	0	0	JALCUH000000000	SRX12655143	7,251
UW_MP_ACT1_2		CSTR	97	0.9961	126			Illumina NovaSeq S4	99.16	0	2.183	18	0.2021	58.0	45	1	0	0	JALCLX000000000	SRX12655187	366
UW_MP_ACT1_3		CSTR	72	0.9998	125			Illumina NovaSeq S4	98.74	0	2.088	19	0.2134	58.0	45	1	0	0	JALCTV000000000	SRX12655142	5,499
UW_MP_ACT1_4		CSTR	84	0.9999	121			Illumina NovaSeq S4	97.9	0.42	1.884	52	0.0475	58.3	42	0	0	0	JALCUR000000000	SRX12655158	3,985
UW_MP_ACT2_1	ACT2	CSTR	126		121	d__Bacteria;p__Actinobacteriota;c__Actinomycetia;o__Actinomycetales;f__Actinomycetaceae;g__Pauljensenia;s__	NA	Illumina NovaSeq S4	96.68	4.1	2.988	40	0.1187	70.5	48	0	0	0	JALCMY000000000	SRX12655887	5,173
UW_MP_ACT2_2		CSTR	168	0.9997	120			Illumina NovaSeq S4	96.26	2.96	2.797	33	0.1295	70.7	45	0	0	0	JALCNR000000000	SRX12655961	2,825
UW_MP_ACT2_3		CSTR	97	0.9977	114			Illumina NovaSeq S4	92.18	3.79	2.437	47	0.0667	71.2	42	0	0	0	JALCLW000000000	SRX12655187	23,464
UW_MP_ACT2_4		CSTR	108	0.9987	114			Illumina NovaSeq S4	90.96	3.79	2.374	48	0.0935	71.2	40	0	0	0	JALCMH000000000	SRX12655904	27,582
UW_MP_ACT2_5		CSTR	120	0.9985	106			Illumina NovaSeq S4	84.55	3.95	2.26	59	0.0512	71.2	39	0	0	0	JALCMQ000000000	SRX12655888	5,493
UW_MP_ACT2_6		CSTR	192	0.9996	99			Illumina NovaSeq S4	77.38	2.61	1.811	77	0.0286	71.4	31	0	0	0	JALCOD000000000	SRX12655976	1,290
UW_MP_ACUT1_1	ACUT1	USB	42		129	d__Bacteria;p__Firmicutes_A;c__Clostridia;o__Oscillospirales;f__Acutalibacteraceae;g__UBA4871;s__	NA	PacBio Sequel II	98.66	0	2.467	2	1.8227	50.6	57	3	3	3	JAKVOA000000000	SAMN18243454	3
UW_MP_ACUT2_1	ACUT2	CSTR	78		113	d__Bacteria;p__Firmicutes_A;c__Clostridia;o__Oscillospirales;f__Acutalibacteraceae;g__UBA4871;s__UBA4871 sp902809935	GCF_902809935.1	Illumina NovaSeq S4	90.13	0.34	1.979	74	0.0353	42.3	41	3	0	0	JALCUG000000000	SRX12655143	303
UW_MP_ACUT2_2		CSTR	72	0.9995	103			Illumina NovaSeq S4	81	1.81	1.835	92	0.0247	42.6	38	3	0	2	JALCTU000000000	SRX12655142	263
UW_MP_ACUT2_3		CSTR	84	0.9996	103			Illumina NovaSeq S4	81.71	0.56	1.732	95	0.0197	42.6	30	3	0	1	JALCUQ000000000	SRX12655158	158
UW_MP_ACUT3_1	ACUT3	CSTR	36		112	d__Bacteria;p__Firmicutes_A;c__Clostridia;o__Oscillospirales;f__Acutalibacteraceae;g__Ruminococcus_E;s__Ruminococcus_E sp902766885	GCA_902766885.1	Illumina NovaSeq S4	90.16	0.67	1.962	78	0.0299	41.6	33	2	1	1	JALCSH000000000	SRX12655159	138
UW_MP_ACUT4_1	ACUT4	USB	63		105	d__Bacteria;p__Firmicutes_A;c__Clostridia;o__Oscillospirales;f__Acutalibacteraceae;g__UBA4871;s__	NA	Illumina NovaSeq S4	82.21	0	1.74	48	0.0546	48.9	32	1	1	1	JAKVJJ000000000	SRX12655160	142
UW_MP_AGRLAC1_1	AGRLAC1	USB	15		125	d__Bacteria;p__Firmicutes;c__Bacilli;o__Lactobacillales;f__Lactobacillaceae;g__Agrilactobacillus;s__	GCF_001436375.1	Illumina NovaSeq S4	99.48	1.05	3.344	25	0.2093	45.0	34	3	0	1	JAKVKU000000000	SRX12655988	494
UW_MP_ANA1_1	ANA1	CSTR	60		131	d__Bacteria;p__Firmicutes_A;c__Clostridia;o__Peptostreptococcales;f__Anaerovoracaceae;g__Eubacterium_T;s__	GCF_900766045.1	PacBio Sequel II	100	0	2.563	1	2.5632	49.8	55	3	3	3	JAKVOJ000000000	SAMN18243139	7
UW_MP_ANA1_2		CSTR	78	0.9997	112			Illumina NovaSeq S4	89.36	0	2.002	56	0.0453	50.3	31	1	0	0	JALCUF000000000	SRX12655143	408
UW_MP_ANA2_1	ANA2	CSTR	12		111	d__Bacteria;p__Firmicutes_A;c__Clostridia;o__Peptostreptococcales;f__Anaerovoracaceae;g__;s__	NA	Illumina NovaSeq S4	89.61	0.35	2.33	70	0.0395	48.6	48	2	1	1	JALCRY000000000	SRX12654482	281
UW_MP_ANA3_1	ANA3	CSTR	54		98	d__Bacteria;p__Firmicutes_A;c__Clostridia;o__Peptostreptococcales;f__Anaerovoracaceae;g__Eubacterium_T;s__Eubacterium_T saphenum_A	GCA_004555725.1	Illumina NovaSeq S4	76.9	0	1.867	92	0.0244	50.1	28	1	0	0	JALCTF000000000	SRX12654502	209
UW_MP_ATO1_1	ATO1	CSTR	97		130	d__Bacteria;p__Actinobacteriota;c__Coriobacteriia;o__Coriobacteriales;f__Atopobiaceae;g__;s__	NA	Illumina NovaSeq S4	100	0	2.025	3	1.1499	65.2	47	1	1	1	JALCLV000000000	SRX12655187	557
UW_MP_ATO1_2		CSTR	78	1.0000	130			Illumina NovaSeq S4	100	0	2.008	3	1.1446	65.3	47	1	1	1	JALCUE000000000	SRX12655143	410
UW_MP_ATO1_3		CSTR	84	1.0000	128			Illumina NovaSeq S4	100	0	2.026	6	0.3687	65.2	48	1	1	1	JALCUP000000000	SRX12655158	327
UW_MP_ATO2_1	ATO2	USB	42		129	d__Bacteria;p__Actinobacteriota;c__Coriobacteriia;o__Coriobacteriales;f__Atopobiaceae;g__;s__	NA	PacBio Sequel II	97.58	0	2.276	1	2.276	65.2	46	1	1	1	JAKVOB000000000	SAMN18243454	9
UW_MP_ATO2_2		USB	77	0.9994	125			Illumina NovaSeq S4	99.19	0	2.104	28	0.1313	65.4	47	0	0	0	JAKVIT000000000	SRX12656338	1,417
UW_MP_ATO2_3		USB	56	0.9994	124			Illumina NovaSeq S4	99.19	0	2.121	27	0.1037	65.4	48	0	0	0	JAKVJX000000000	SRX12656290	1,558
UW_MP_ATO2_4		USB	28	0.9993	122			Illumina NovaSeq S4	97.58	1.61	2.113	40	0.0603	65.4	40	0	0	0	JAKVKL000000000	SRX12656283	4,342
UW_MP_ATO2_5		USB	105	0.9992	120			Illumina NovaSeq S4	94.84	0.4	2.108	38	0.0644	65.3	48	0	0	0	JALCCP000000000	SRX12656339	284
UW_MP_ATO2_6		USB	63	0.9994	117			Illumina NovaSeq S4	94.35	0.6	1.85	67	0.0359	65.7	41	0	0	0	JAKVJK000000000	SRX12656289	814
UW_MP_ATO2_7		USB	176	0.9993	111			Illumina NovaSeq S4	87.9	1.01	1.894	72	0.0309	65.6	44	1	0	1	JALCDI000000000	SRX12657171	181
UW_MP_ATO3_1	ATO3	CSTR	108		124	d__Bacteria;p__Actinobacteriota;c__Coriobacteriia;o__Coriobacteriales;f__Atopobiaceae;g__Olsenella_B;s__	NA	Illumina NovaSeq S4	97.98	0	2.454	28	0.1431	61.8	46	0	0	0	JALCMG000000000	SRX12655904	20,248
UW_MP_ATO3_2		CSTR	126	0.9996	123			Illumina NovaSeq S4	97.58	0	2.434	29	0.1341	61.9	46	0	0	0	JALCMX000000000	SRX12655887	9,939
UW_MP_ATO3_3		CSTR	146	0.9997	122			Illumina NovaSeq S4	96.77	0	2.474	46	0.0912	61.7	47	0	0	0	JALCNJ000000000	SRX12655903	369
UW_MP_ATO3_4		CSTR	192	0.9994	121			Illumina NovaSeq S4	97.58	0.81	2.303	64	0.043	61.9	44	0	0	0	JALCOC000000000	SRX12655976	13,183
UW_MP_ATO3_5		CSTR	216	0.9933	121			PacBio Sequel II	92	1.61	2.589	5	0.5243	62.0	37	2	2	2	JAKVOH000000000	SAMN18243112	5
UW_MP_ATO3_6		CSTR	60	0.9883	113			PacBio Sequel II	85.48	0.81	2.449	10	0.2981	62.1	39	2	2	2	JAKVOK000000000	SAMN18243139	94
UW_MP_ATO3_7		CSTR	72	0.9931	111			Illumina NovaSeq S4	88.69	0.81	1.779	79	0.0265	62.0	40	0	0	0	JALCTT000000000	SRX12655142	46,660
UW_MP_ATO3_8		CSTR	97	0.9914	111			Illumina NovaSeq S4	88.44	0	1.79	78	0.0277	62.0	45	0	0	0	JALCLU000000000	SRX12655187	13,530
UW_MP_ATO3_9		CSTR	66	0.9932	110			Illumina NovaSeq S4	87.92	0	1.734	84	0.0238	62.1	38	0	0	0	JALCTN000000000	SRX12654503	55,155
UW_MP_ATO3_10		CSTR	84	0.9916	109			Illumina NovaSeq S4	86.42	0	1.757	75	0.0308	61.9	39	1	0	0	JALCUO000000000	SRX12655158	46,496
UW_MP_ATO3_11		CSTR	78	0.9918	107			Illumina NovaSeq S4	84.41	0	1.834	82	0.0288	61.9	39	0	0	0	JALCUD000000000	SRX12655143	35,365
UW_MP_ATO3_12		CSTR	49	0.9930	105			Illumina NovaSeq S4	82.53	0.81	1.665	81	0.0223	62.1	37	1	0	1	JALCSX000000000	SRX12654484	25,716
UW_MP_ATO3_13		CSTR	54	0.9930	104			Illumina NovaSeq S4	81.85	0	1.625	79	0.0231	62.1	38	0	0	0	JALCTE000000000	SRX12654502	36,563
UW_MP_ATO4_1	ATO4	CSTR	44		120	d__Bacteria;p__Actinobacteriota;c__Coriobacteriia;o__Coriobacteriales;f__Atopobiaceae;g__;s__	NA	PacBio Sequel II	93.71	4.95	2.611	33	0.1367	66.3	49	1	1	1	JAKVON000000000	SAMN18243037	2
UW_MP_ATO5_1	ATO5	CSTR	24		115	d__Bacteria;p__Actinobacteriota;c__Coriobacteriia;o__Coriobacteriales;f__Atopobiaceae;g__UBA7748;s__UBA7748 sp900314535	GCA_900314535.1	PacBio Sequel II	91.13	0.81	2.448	35	0.1065	60.5	46	4	3	4	JAKVOX000000000	SAMN18243588	3
UW_MP_ATO5_2		CSTR	12	0.9967	99			Illumina NovaSeq S4	77.3	0	1.494	72	0.0235	60.5	37	0	0	0	JALCRX000000000	SRX12654482	3,246
UW_MP_ATO6_1	ATO6	CSTR	36		112	d__Bacteria;p__Actinobacteriota;c__Coriobacteriia;o__Coriobacteriales;f__Atopobiaceae;g__Olsenella_B;s__Olsenella_B sp900119625	GCF_900119625.1	Illumina NovaSeq S4	84.95	0	1.818	17	0.5024	62.7	46	0	0	0	JALCSG000000000	SRX12654483	9,573
UW_MP_BACIL1_1	BACIL1	CSTR	24		128	d__Bacteria;p__Firmicutes;c__Bacilli;o__RFN20;f__CAG-826;g__UBA4951;s__UBA4951 sp002397465	GCA_002397465.1	PacBio Sequel II	97.75	1.12	1.628	1	1.6276	48.4	48	1	1	1	JAKVOY000000000	SAMN18243588	2
UW_MP_BACIL1_2		USB	56	0.9982	107			Illumina NovaSeq S4	85.64	1.12	1.102	51	0.0242	48.7	32	0	1	0	JAKVJY000000000	SRX12656290	96
UW_MP_BACIL1_3		CSTR	12	0.9996	105			Illumina NovaSeq S4	83.15	1.4	1.051	46	0.029	48.7	31	1	2	1	JALCRW000000000	SRX12654482	151
UW_MP_BACIL1_4		USB	77	0.9977	103			Illumina NovaSeq S4	81.39	1.12	1.238	50	0.0319	48.8	38	1	1	2	JAKVIU000000000	SRX12656338	198
UW_MP_BACIL1_5		USB	63	0.9976	101			Illumina NovaSeq S4	78.65	0.56	1.146	52	0.0235	48.7	36	1	1	1	JAKVJL000000000	SRX12656289	153
UW_MP_BACIL2_1	BACIL2	CSTR	44		128	d__Bacteria;p__Firmicutes;c__Bacilli;o__RFN20;f__CAG-826;g__UBA7706;s__	NA	PacBio Sequel II	98.31	0	1.497	2	1.482	37.9	45	1	1	1	JAKVOO000000000	SAMN18243037	1
UW_MP_BACIL3_1	BACIL3	CSTR	12		123	d__Bacteria;p__Firmicutes;c__Bacilli;o__RFN20;f__CAG-288;g__UBA6879;s__	NA	Illumina NovaSeq S4	97.75	1.5	2.094	20	0.1663	43.3	47	2	0	0	JALCRV000000000	SRX12654482	289
UW_MP_BACIL4_1	BACIL4	USB	148		110	d__Bacteria;p__Firmicutes;c__Bacilli;o__RFN20;f__CAG-826;g__UBA4951;s__	NA	Illumina NovaSeq S4	85.39	0.05	1.591	15	0.2379	52.4	48	1	1	1	JALCDB000000000	SRX12657082	375
UW_MP_BACIL5_1	BACIL5	USB	63		108	d__Bacteria;p__Firmicutes;c__Bacilli;o__RFN20;f__CAG-826;g__UBA7706;s__	NA	Illumina NovaSeq S4	85.62	0.05	1.236	57	0.0258	38.0	39	0	1	0	JAKVJM000000000	SRX12656289	173
UW_MP_BACIL5_2		USB	56	0.9996	107			Illumina NovaSeq S4	85.96	0.05	1.318	63	0.0232	37.9	41	0	0	0	JAKVJZ000000000	SRX12656290	156
UW_MP_BACIL6_1	BACIL6	CSTR	49		107	d__Bacteria;p__Firmicutes;c__Bacilli;o__RFN20;f__CAG-826;g__UBA4951;s__	NA	Illumina NovaSeq S4	79.78	0	1.29	4	0.5045	54.5	46	1	1	1	JALCSW000000000	SRX12654484	230
UW_MP_BACIL7_1	BACIL7	USB	42		106	d__Bacteria;p__Firmicutes;c__Bacilli;o__RFN20;f__CAG-288;g__UBA7642;s__	NA	PacBio Sequel II	81.09	4.49	1.11	17	0.0801	49.3	28	2	2	2	JAKVOC000000000	SAMN18243454	1
UW_MP_BACIL8_1	BACIL8	CSTR	12		102	d__Bacteria;p__Firmicutes;c__Bacilli;o__RF39;f__UBA660;g__CAG-451;s__CAG-451 sp000438295	GCA_000438295.1	Illumina NovaSeq S4	80.34	1.69	1.073	36	0.0438	27.9	31	2	0	0	JALCRU000000000	SRX12654482	1,424
UW_MP_BACTE1_1	BACTE1	CSTR	44		126	d__Bacteria;p__Bacteroidota;c__Bacteroidia;o__Bacteroidales;f__UBA932;g__RC9;s__RC9 sp900316045	GCA_900316045.1	PacBio Sequel II	96.9	1.19	3.242	4	0.8803	49.0	44	4	3	3	JAKVOP000000000	SAMN18243037	9
UW_MP_BACTE1_2		CSTR	49	0.9998	99			Illumina NovaSeq S4	77.03	1.19	2.343	81	0.0347	49.0	37	3	0	0	JALCSV000000000	SRX12654484	5,271
UW_MP_BACTE2_1	BACTE2	CSTR	126		122	d__Bacteria;p__Bacteroidota;c__Bacteroidia;o__Bacteroidales;f__UBA932;g__RC9;s__	NA	Illumina NovaSeq S4	95.24	1.19	2.426	16	0.338	43.7	41	1	1	0	JALCMW000000000	SRX12655887	701
UW_MP_BACTE2_2		CSTR	108	0.9999	97			Illumina NovaSeq S4	75.37	0.48	1.551	84	0.0231	44.0	28	0	1	1	JALCMF000000000	SRX12655904	112
UW_MP_BACTE3_1	BACTE3	CSTR	108		120	d__Bacteria;p__Bacteroidota;c__Bacteroidia;o__Bacteroidales;f__Bacteroidaceae;g__Bacteroides;s__	NA	Illumina NovaSeq S4	94.42	1.63	4.26	42	0.1502	40.0	50	0	0	0	JALCME000000000	SRX12655904	627
UW_MP_BACTE3_2		CSTR	97	0.9995	97			Illumina NovaSeq S4	75.56	1.67	2.979	166	0.0182	39.4	35	0	0	0	JALCLT000000000	SRX12655187	297
UW_MP_BACTE4_1	BACTE4	CSTR	120		118	d__Bacteria;p__Bacteroidota;c__Bacteroidia;o__Bacteroidales;f__UBA932;g__UBA1232;s__	NA	Illumina NovaSeq S4	95	0.95	2.367	56	0.0637	41.8	39	1	3	1	JALCMP000000000	SRX12655888	208
UW_MP_BACTE5_1	BACTE5	CSTR	36		117	d__Bacteria;p__Bacteroidota;c__Bacteroidia;o__Bacteroidales;f__UBA932;g__RC9;s__RC9 sp900316045	GCA_900316045.1	Illumina NovaSeq S4	91.43	0.95	2.926	27	0.192	48.3	40	0	1	1	JALCSF000000000	SRX12654483	7,427
UW_MP_BACTE5_2		CSTR	49	0.9987	100			Illumina NovaSeq S4	77.48	0.95	2.445	66	0.0431	48.3	31	0	2	0	JALCSU000000000	SRX12654484	3,692
UW_MP_BACTE6_1	BACTE6	CSTR	49		117	d__Bacteria;p__Bacteroidota;c__Bacteroidia;o__Bacteroidales;f__UBA932;g__;s__	NA	Illumina NovaSeq S4	91.79	0	2.185	23	0.1385	48.4	42	1	2	0	JALCST000000000	SRX12654484	425
UW_MP_BACTE6_2		CSTR	36	0.9999	115			Illumina NovaSeq S4	90.48	0.48	2.089	34	0.0966	48.4	36	1	3	1	JALCSE000000000	SRX12654483	403
UW_MP_BACTE6_3		CSTR	54	0.9999	103			Illumina NovaSeq S4	82.13	0	1.744	92	0.0217	48.8	29	0	1	0	JALCTD000000000	SRX12654502	129
UW_MP_BACTE7_1	BACTE7	CSTR	24		117	d__Bacteria;p__Bacteroidota;c__Bacteroidia;o__Bacteroidales;f__P3;g__;s__	NA	PacBio Sequel II	91.84	0	1.881	23	0.1506	29.7	34	1	2	1	JAKVOZ000000000	SAMN18243588	1
UW_MP_BACTE8_1	BACTE8	CSTR	12		101	d__Bacteria;p__Bacteroidota;c__Bacteroidia;o__Bacteroidales;f__UBA932;g__RC9;s__RC9 sp000432515	GCA_000432515.1	Illumina NovaSeq S4	80.01	1.9	2.392	109	0.0251	49.2	31	1	2	0	JALCRT000000000	SRX12654482	346
UW_MP_BIF1_1	BIF1	CSTR	49		130	d__Bacteria;p__Actinobacteriota;c__Actinomycetia;o__Actinomycetales;f__Bifidobacteriaceae;g__Bifidobacterium;s__Bifidobacterium breve	GCF_001025175.1	Illumina NovaSeq S4	100	0	2.182	4	1.1285	58.8	53	2	0	0	JALCSS000000000	SRX12654484	731
UW_MP_BIF1_2		CSTR	54	1.0000	127			Illumina NovaSeq S4	99.92	0	2.179	10	0.3004	58.8	53	2	1	0	JALCTC000000000	SRX12654502	370
UW_MP_BIF2_1	BIF2	USB	42		130	d__Bacteria;p__Actinobacteriota;c__Actinomycetia;o__Actinomycetales;f__Bifidobacteriaceae;g__Bifidobacterium;s__Bifidobacterium tibiigranuli	GCF_009371885.1	PacBio Sequel II	99.12	4.36	3.1	3	3.0762	60.6	50	2	2	2	JAKVOD000000000	SAMN18243454	27
UW_MP_BIF2_2		USB	56	0.9999	122			Illumina NovaSeq S4	97.39	3.14	2.673	16	0.2331	60.5	45	2	2	2	JAKVKB000000000	SRX12656290	27,953
UW_MP_BIF2_3		USB	77	0.9999	122			Illumina NovaSeq S4	97.39	3.14	2.654	19	0.2026	60.5	45	0	0	0	JAKVIV000000000	SRX12656338	16,783
UW_MP_BIF2_4		USB	105	0.9883	120			Illumina NovaSeq S4	95.12	3.37	2.656	24	0.1768	60.5	42	0	0	0	JALCCO000000000	SRX12656339	19,505
UW_MP_BIF2_5		USB	126	0.9893	118			Illumina NovaSeq S4	95.74	3.83	2.535	57	0.0713	60.6	40	0	0	0	JALCCS000000000	SRX12656446	25,167
UW_MP_BIF2_6		CSTR	146	0.9952	117			Illumina NovaSeq S4	91.94	1.48	2.326	35	0.1149	60.6	39	0	0	0	JALCNI000000000	SRX12655903	11,592
UW_MP_BIF2_7		CSTR	126	0.9918	112			Illumina NovaSeq S4	88.7	2.39	2.316	48	0.0776	60.7	37	0	0	0	JALCMV000000000	SRX12655887	2,983
UW_MP_BIF2_8		USB	28	0.9992	103			Illumina NovaSeq S4	80.75	4.28	2.398	47	0.074	60.5	42	0	0	0	JAKVKM000000000	SRX12656283	3,617
UW_MP_BIF2_9		USB	176	0.9929	103			Illumina NovaSeq S4	78.95	1.7	1.894	40	0.0758	60.7	32	0	0	0	JALCDG000000000	SRX12657171	26,915
UW_MP_BIF2_10		USB	148	0.9934	100			Illumina NovaSeq S4	77.81	2.31	1.878	55	0.048	61.1	28	0	0	0	JALCCZ000000000	SRX12657082	34,329
UW_MP_BIF3_1	BIF3	USB	197		129	d__Bacteria;p__Actinobacteriota;c__Actinomycetia;o__Actinomycetales;f__Bifidobacteriaceae;g__Bifidobacterium;s__	NA	PacBio Sequel II	98.86	0.91	2.342	1	2.3423	48.8	46	2	2	2	JALCLM000000000	SRX12729151	9
UW_MP_BIF4_1	BIF4	CSTR	192		127	d__Bacteria;p__Actinobacteriota;c__Actinomycetia;o__Actinomycetales;f__Bifidobacteriaceae;g__Bifidobacterium;s__Bifidobacterium crudilactis	GCF_000738005.1	Illumina NovaSeq S4	98.32	1.82	2.253	3	1.3824	57.8	48	0	1	0	JALCOB000000000	SRX12655976	670
UW_MP_BIF5_1	BIF5	CSTR	72		124	d__Bacteria;p__Actinobacteriota;c__Actinomycetia;o__Actinomycetales;f__Bifidobacteriaceae;g__Bifidobacterium;s__Bifidobacterium crudilactis	GCF_000738005.1	Illumina NovaSeq S4	99.23	1.93	2.313	25	0.1431	57.5	50	2	2	2	JALCTS000000000	SRX12655142	1,206
UW_MP_BIF5_2		CSTR	66	1.0000	117			Illumina NovaSeq S4	93.11	1.44	2.062	49	0.0643	57.5	42	0	0	0	JALCTM000000000	SRX12654503	666
UW_MP_BIF6_1	BIF6	CSTR	78		124	d__Bacteria;p__Actinobacteriota;c__Actinomycetia;o__Actinomycetales;f__Bifidobacteriaceae;g__Bifidobacterium;s__Bifidobacterium psychraerophilum	GCF_000741705.1	Illumina NovaSeq S4	98.41	1.97	2.494	22	0.218	59.2	46	0	0	0	JALCUB000000000	SRX12655143	1,025
UW_MP_BIF6_2		CSTR	84	0.9998	122			Illumina NovaSeq S4	97.05	2.73	2.466	34	0.1047	59.1	46	0	0	0	JALCUN000000000	SRX12655158	283
UW_MP_BIF6_3		CSTR	146	0.9999	120			Illumina NovaSeq S4	96.44	1.67	2.401	44	0.0765	59.1	44	0	0	0	JALCNH000000000	SRX12655903	220
UW_MP_BIF6_4		CSTR	108	1.0000	105			Illumina NovaSeq S4	82.86	0.67	2.142	73	0.034	59.0	42	0	0	0	JALCMC000000000	SRX12655904	487
UW_MP_BIF7_1	BIF7	USB	126		124	d__Bacteria;p__Actinobacteriota;c__Actinomycetia;o__Actinomycetales;f__Bifidobacteriaceae;g__Bifidobacterium;s__Bifidobacterium crudilactis	GCF_000738005.1	Illumina NovaSeq S4	99.17	1.67	2.32	16	0.2279	57.7	45	0	0	0	JALCCR000000000	SRX12656446	3,089
UW_MP_BIF8_1	BIF8	CSTR	168		123	d__Bacteria;p__Actinobacteriota;c__Actinomycetia;o__Actinomycetales;f__Bifidobacteriaceae;g__Bifidobacterium;s__	NA	Illumina NovaSeq S4	98.57	2.2	2.367	33	0.0991	61.8	44	0	0	0	JALCNP000000000	SRX12655961	422
UW_MP_BIF8_2		CSTR	192	0.9994	115			Illumina NovaSeq S4	92.02	1.29	1.991	61	0.0429	62.4	38	1	0	1	JALCOA000000000	SRX12655976	2,179
UW_MP_BIF9_1	BIF9	CSTR	84		123	d__Bacteria;p__Actinobacteriota;c__Actinomycetia;o__Actinomycetales;f__Bifidobacteriaceae;g__Bifidobacterium;s__Bifidobacterium tibiigranuli	GCF_009371885.1	Illumina NovaSeq S4	94.82	2.58	2.669	10	0.7116	60.6	46	0	0	0	JALCUM000000000	SRX12655158	1,485
UW_MP_BIF9_2		CSTR	78	1.0000	122			Illumina NovaSeq S4	95.73	2.5	2.555	22	0.2925	60.8	45	0	0	0	JALCUA000000000	SRX12655143	1,512
UW_MP_BIF9_3		CSTR	97	0.9998	107			Illumina NovaSeq S4	84.22	2.5	2.23	61	0.0601	60.7	37	0	0	0	JALCLR000000000	SRX12655187	1,353
UW_MP_BIF10_1	BIF10	USB	197		122	d__Bacteria;p__Actinobacteriota;c__Actinomycetia;o__Actinomycetales;f__Bifidobacteriaceae;g__Bifidobacterium;s__Bifidobacterium crudilactis	GCF_000738005.1	PacBio Sequel II	92.41	1.21	2.062	1	2.0624	57.7	42	0	0	0	JALCLO000000000	SRX12729151	34
UW_MP_BIF11_1	BIF11	CSTR	120		121	d__Bacteria;p__Actinobacteriota;c__Actinomycetia;o__Actinomycetales;f__Bifidobacteriaceae;g__Bifidobacterium;s__Bifidobacterium tibiigranuli	GCF_009371885.1	Illumina NovaSeq S4	94.88	2.88	2.706	17	0.3076	60.6	42	0	0	0	JALCMO000000000	SRX12655888	13,270
UW_MP_BIF11_2		CSTR	108	0.9996	118			Illumina NovaSeq S4	93.19	2.88	2.543	26	0.1275	60.5	41	0	0	0	JALCMD000000000	SRX12655904	6,115
UW_MP_BIF11_3		CSTR	168	0.9957	102			Illumina NovaSeq S4	79.42	2.12	2.297	55	0.0569	60.4	42	0	0	0	JALCNQ000000000	SRX12655961	12,520
UW_MP_BIF12_1	BIF12	USB	126		118	d__Bacteria;p__Actinobacteriota;c__Actinomycetia;o__Actinomycetales;f__Bifidobacteriaceae;g__Bifidobacterium;s__	NA	Illumina NovaSeq S4	94.72	2.27	2.15	53	0.0569	60.7	41	2	0	0	JALCCV000000000	SRX12656446	327
UW_MP_BIF12_2		USB	56	0.9999	117			Illumina NovaSeq S4	92.34	2.12	2.131	34	0.141	60.7	46	1	1	1	JAKVKA000000000	SRX12656290	337
UW_MP_BIF13_1	BIF13	USB	21		114	d__Bacteria;p__Actinobacteriota;c__Actinomycetia;o__Actinomycetales;f__Bifidobacteriaceae;g__Bifidobacterium;s__	NA	Illumina NovaSeq S4	88.64	1.36	2.037	24	0.1459	48.6	41	0	0	0	JAKVKQ000000000	SRX12655989	976
UW_MP_BIF13_2		USB	15	1.0000	110			Illumina NovaSeq S4	85.71	1.36	1.999	25	0.1141	48.6	38	0	0	0	JAKVKV000000000	SRX12655988	992
UW_MP_BIF14_1	BIF14	USB	148		112	d__Bacteria;p__Actinobacteriota;c__Actinomycetia;o__Actinomycetales;f__Bifidobacteriaceae;g__Bifidobacterium;s__Bifidobacterium subtile	GCF_000741775.1	Illumina NovaSeq S4	89.48	3.03	2.186	50	0.0601	61.2	39	0	1	0	JALCDA000000000	SRX12657082	2,259
UW_MP_BIF14_2		USB	126	0.9975	106			Illumina NovaSeq S4	83.66	1.06	2.076	70	0.0367	61.3	33	0	0	0	JALCCU000000000	SRX12656446	2,424
UW_MP_BIF14_3		USB	176	0.9995	100			Illumina NovaSeq S4	77.67	1.36	1.81	54	0.0516	61.2	36	0	1	0	JALCDH000000000	SRX12657171	975
UW_MP_BIF15_1	BIF15	USB	197		111	d__Bacteria;p__Actinobacteriota;c__Actinomycetia;o__Actinomycetales;f__Bifidobacteriaceae;g__Bifidobacterium;s__Bifidobacterium crudilactis	GCF_000738005.1	PacBio Sequel II	84.03	0.98	1.751	5	0.4795	57.6	39	0	0	0	JALCLN000000000	SRX12729151	9
UW_MP_BIF16_1	BIF16	CSTR	97		111	d__Bacteria;p__Actinobacteriota;c__Actinomycetia;o__Actinomycetales;f__Bifidobacteriaceae;g__Bifidobacterium;s__Bifidobacterium crudilactis	GCF_000738005.1	Illumina NovaSeq S4	90.81	2.23	1.957	84	0.0266	57.9	43	1	0	1	JALCLS000000000	SRX12655187	322
UW_MP_BIF17_1	BIF17	USB	15		111	d__Bacteria;p__Actinobacteriota;c__Actinomycetia;o__Actinomycetales;f__Bifidobacteriaceae;g__Bifidobacterium;s__Bifidobacterium minimum	GCF_000741645.1	Illumina NovaSeq S4	89.08	0.66	1.488	68	0.0271	63.2	38	0	0	0	JAKVKW000000000	SRX12655988	243
UW_MP_BIF18_1	BIF18	CSTR	78		109	d__Bacteria;p__Actinobacteriota;c__Actinomycetia;o__Actinomycetales;f__Bifidobacteriaceae;g__Bifidobacterium;s__Bifidobacterium thermophilum	GCF_000771265.1	Illumina NovaSeq S4	87.44	0.95	1.802	63	0.0375	60.5	32	1	0	1	JALCUC000000000	SRX12655143	9,522
UW_MP_BIF19_1	BIF19	USB	126		105	d__Bacteria;p__Actinobacteriota;c__Actinomycetia;o__Actinomycetales;f__Bifidobacteriaceae;g__Bifidobacterium;s__	NA	Illumina NovaSeq S4	81.29	1.21	1.761	22	0.1391	58.8	40	0	1	0	JALCCT000000000	SRX12656446	509
UW_MP_BUL1_1	BUL1	USB	56		120	d__Bacteria;p__Firmicutes;c__Bacilli;o__Erysipelotrichales;f__Erysipelotrichaceae;g__Bulleidia;s__	NA	Illumina NovaSeq S4	96.05	0.79	2.075	40	0.0737	45.2	38	1	1	1	JAKVKC000000000	SRX12656290	4,421
UW_MP_BUL1_2		USB	77	1.0000	118			Illumina NovaSeq S4	94.15	0.79	2.163	40	0.0737	44.9	41	0	0	0	JAKVIW000000000	SRX12656338	3,071
UW_MP_BUL1_3		USB	63	1.0000	116			Illumina NovaSeq S4	92.24	0.32	2.041	36	0.0751	45.2	41	0	0	0	JAKVJN000000000	SRX12656289	4,322
UW_MP_BUL1_4		USB	42	0.9990	106			PacBio Sequel II	78.01	0.79	2.313	7	0.4212	44.9	42	1	1	1	JAKVOE000000000	SAMN18243454	8
UW_MP_BUL2_1	BUL2	CSTR	66		116	d__Bacteria;p__Firmicutes;c__Bacilli;o__Erysipelotrichales;f__Erysipelotrichaceae;g__Bulleidia;s__Bulleidia massiliensis_B	GCF_900290205.1	Illumina NovaSeq S4	94.29	0.32	2.418	54	0.0627	50.1	43	0	0	0	JALCTL000000000	SRX12654503	3,619
UW_MP_BUL3_1	BUL3	USB	56		103	d__Bacteria;p__Firmicutes;c__Bacilli;o__Erysipelotrichales;f__Erysipelotrichaceae;g__Bulleidia;s__	NA	Illumina NovaSeq S4	81.19	1.27	1.989	85	0.0305	48.8	35	1	0	0	JAKVKD000000000	SRX12656290	207
UW_MP_BUL3_2		USB	63	0.9998	100			Illumina NovaSeq S4	77.78	0.32	1.731	80	0.0275	49.1	31	0	0	0	JAKVJO000000000	SRX12656289	146
UW_MP_BUL3_3		USB	77	0.9997	99			Illumina NovaSeq S4	77.14	0.32	1.882	82	0.0273	49.0	30	1	0	0	JAKVIX000000000	SRX12656338	161
UW_MP_BURK1_1	BURK1	USB	77		123	d__Bacteria;p__Proteobacteria;c__Gammaproteobacteria;o__Burkholderiales;f__Burkholderiaceae;g__Kerstersia;s__Kerstersia gyiorum	GCF_004216755.1	Illumina NovaSeq S4	98.33	0	3.756	67	0.0811	62.6	49	1	1	1	JAKVIY000000000	SRX12656338	373
UW_MP_BURK1_2		USB	105	0.9998	121			Illumina NovaSeq S4	96.68	0.54	3.569	73	0.0612	62.6	43	0	0	0	JALCCN000000000	SRX12656339	444
UW_MP_BURK2_1	BURK2	CSTR	146		120	d__Bacteria;p__Proteobacteria;c__Gammaproteobacteria;o__Burkholderiales;f__Burkholderiaceae;g__Achromobacter;s__Achromobacter ruhlandii	GCF_902859695.1	Illumina NovaSeq S4	97.25	0.23	5.869	141	0.058	67.9	52	2	1	1	JALCNG000000000	SRX12655903	588
UW_MP_BURK3_1	BURK3	CSTR	108		116	d__Bacteria;p__Proteobacteria;c__Gammaproteobacteria;o__Burkholderiales;f__Burkholderiaceae;g__Mesosutterella;s__Mesosutterella multiformis	GCF_003402575.1	Illumina NovaSeq S4	93.33	0	1.739	42	0.0581	57.9	45	2	1	0	JALCMB000000000	SRX12655904	930
UW_MP_BURK4_1	BURK4	USB	63		101	d__Bacteria;p__Proteobacteria;c__Gammaproteobacteria;o__Burkholderiales;f__Burkholderiaceae;g__Alcaligenes;s__Alcaligenes aquatilis	GCF_003076515.1	Illumina NovaSeq S4	79.99	0.18	3.138	131	0.0268	56.1	40	0	0	0	JAKVJP000000000	SRX12656289	1,134
UW_MP_CARN1_1	CARN1	CSTR	168		116	d__Bacteria;p__Firmicutes;c__Bacilli;o__Lactobacillales;f__Carnobacteriaceae;g__Carnobacterium;s__Carnobacterium maltaromaticum	GCF_000744945.1	Illumina NovaSeq S4	95.63	7.19	3.399	79	0.0624	34.4	35	4	0	0	JALCNO000000000	SRX12655961	272
UW_MP_CAUL1_1	CAUL1	USB	77		127	d__Bacteria;p__Proteobacteria;c__Alphaproteobacteria;o__Caulobacterales;f__Caulobacteraceae;g__Brevundimonas;s__Brevundimonas sp002386585	GCA_002386585.1	Illumina NovaSeq S4	100	0.39	3.598	15	0.4877	67.4	49	1	1	1	JAKVIZ000000000	SRX12656338	736
UW_MP_CLOS1_1	CLOS1	CSTR	216		132	d__Bacteria;p__Firmicutes_A;c__Clostridia;o__Clostridiales;f__Clostridiaceae;g__Clostridium_B;s__	NA	PacBio Sequel II	99.83	0	3.567	1	3.5672	35.0	66	7	6	6	JAKVOI000000000	SAMN18243112	10
UW_MP_CLOS1_2		CSTR	126	1.0000	127			Illumina NovaSeq S4	99.83	0	3.434	23	0.3067	34.9	61	4	3	0	JALCMU000000000	SRX12655887	34,895
UW_MP_CLOS1_3		CSTR	120	1.0000	127			Illumina NovaSeq S4	99.83	0	3.385	23	0.2571	34.9	61	4	3	0	JALCMN000000000	SRX12655888	29,082
UW_MP_CLOS1_4		CSTR	146	1.0000	127			Illumina NovaSeq S4	99.83	0	3.381	22	0.2535	34.9	61	4	3	0	JALCNF000000000	SRX12655903	36,752
UW_MP_CLOS1_5		CSTR	192	1.0000	127			Illumina NovaSeq S4	99.83	0	3.39	23	0.2535	34.9	61	4	2	0	JALCNZ000000000	SRX12655976	33,252
UW_MP_CLOS1_6		CSTR	84	1.0000	122			Illumina NovaSeq S4	98.91	0	3.34	87	0.0504	35.0	58	4	3	0	JALCUL000000000	SRX12655158	309
UW_MP_CLOS2_1	CLOS2	USB	197		132	d__Bacteria;p__Firmicutes_A;c__Clostridia;o__Clostridiales;f__Clostridiaceae;g__Clostridium_B;s__Clostridium_B tyrobutyricum	GCF_000359585.1	PacBio Sequel II	99.49	0.23	3.062	1	3.0624	31.0	63	7	6	6	JALCLL000000000	SRX12729151	18
UW_MP_CLOS2_2		USB	77	0.9999	123			Illumina NovaSeq S4	98.11	0.36	3.17	49	0.1032	30.5	59	3	1	0	JAKVJA000000000	SRX12656338	1,461
UW_MP_CLOS2_3		USB	56	0.9998	117			Illumina NovaSeq S4	93.6	0.27	3.01	109	0.0384	30.6	52	3	1	0	JAKVKE000000000	SRX12656290	415
UW_MP_CLOS2_4		USB	63	0.9995	106			Illumina NovaSeq S4	84.24	0.33	2.377	119	0.0242	31.0	43	3	1	0	JAKVJQ000000000	SRX12656289	193
UW_MP_CLOS2_5		USB	148	1.0000	105			Illumina NovaSeq S4	79.59	0.23	2.664	32	0.152	31.0	32	0	0	0	JALCCY000000000	SRX12657082	713
UW_MP_EGG1_1	EGG1	USB	63		113	d__Bacteria;p__Actinobacteriota;c__Coriobacteriia;o__Coriobacteriales;f__Eggerthellaceae;g__UBA5808;s__UBA5808 sp002417975	GCA_002417975.1	Illumina NovaSeq S4	89.85	0.81	1.734	62	0.0349	46.5	41	0	0	0	JAKVJR000000000	SRX12656289	193
UW_MP_EGG1_2		USB	56	0.9997	102			Illumina NovaSeq S4	79.61	0.27	1.656	58	0.0307	46.6	37	0	0	0	JAKVKF000000000	SRX12656290	330
UW_MP_ENTER1_1	ENTER1	CSTR	168		127	d__Bacteria;p__Proteobacteria;c__Gammaproteobacteria;o__Enterobacterales;f__Enterobacteriaceae;g__Ewingella;s__Ewingella americana	GCF_000735345.1	Illumina NovaSeq S4	97.7	0.25	4.882	10	0.7672	53.9	65	3	0	1	JALCNN000000000	SRX12655961	3,203
UW_MP_ENTER1_2		CSTR	192	1.0000	126			Illumina NovaSeq S4	98.36	0.25	4.911	17	0.4431	53.9	66	3	0	1	JALCNY000000000	SRX12655976	1,154
UW_MP_ENTER1_3		CSTR	84	1.0000	126			Illumina NovaSeq S4	98.54	0.27	4.929	24	0.3563	53.8	64	3	1	0	JALCUK000000000	SRX12655158	712
UW_MP_ENTER1_4		CSTR	97	1.0000	126			Illumina NovaSeq S4	97.7	0.25	4.853	14	0.5036	53.9	65	3	0	0	JALCLQ000000000	SRX12655187	1,440
UW_MP_ENTER1_5		CSTR	72	1.0000	122			Illumina NovaSeq S4	97.14	0.41	4.791	59	0.1111	53.9	65	3	0	0	JALCTR000000000	SRX12655142	571
UW_MP_ENTER1_6		CSTR	66	0.9999	119			Illumina NovaSeq S4	93.57	1.18	4.507	42	0.1385	54.0	56	5	0	0	JALCTK000000000	SRX12654503	540
UW_MP_ENTER2_1	ENTER2	USB	148		126	d__Bacteria;p__Proteobacteria;c__Gammaproteobacteria;o__Enterobacterales;f__Enterobacteriaceae;g__Serratia;s__Serratia liquefaciens	GCF_000422085.1	Illumina NovaSeq S4	98.21	0.56	5.147	22	0.479	55.5	75	2	0	0	JALCCX000000000	SRX12657082	1,977
UW_MP_ENTER2_2		CSTR	168	1.0000	126			Illumina NovaSeq S4	98.76	0.45	5.136	26	0.3624	55.5	74	3	0	0	JALCNM000000000	SRX12655961	947
UW_MP_ENTER2_3		USB	126	1.0000	126			Illumina NovaSeq S4	98.21	0.56	5.15	20	0.4788	55.5	74	2	0	0	JALCCQ000000000	SRX12656446	3,786
UW_MP_ENTER2_4		USB	105	1.0000	126			Illumina NovaSeq S4	98.21	0.56	5.151	21	0.3835	55.5	75	2	0	0	JALCCM000000000	SRX12656339	1,995
UW_MP_ENTER2_5		CSTR	0	1.0000	125			Illumina NovaSeq S4	98.21	0.45	5.139	29	0.2433	55.5	74	3	0	0	JALCRN000000000	SRX12654481	886
UW_MP_ENTER2_6		USB	77	1.0000	123			Illumina NovaSeq S4	96.8	1	5.032	37	0.2053	55.6	73	3	1	0	JAKVJB000000000	SRX12656338	619
UW_MP_ENTER2_7		USB	56	1.0000	123			Illumina NovaSeq S4	97.67	0.62	5.06	62	0.1217	55.6	72	2	0	0	JAKVKG000000000	SRX12656290	513
UW_MP_ENTER2_8		CSTR	146	1.0000	122			Illumina NovaSeq S4	94.64	0.45	5.068	17	0.4056	55.5	71	2	0	0	JALCNE000000000	SRX12655903	4,113
UW_MP_ENTER2_9		CSTR	192	1.0000	118			Illumina NovaSeq S4	95.04	1.49	4.95	106	0.0672	55.6	73	3	1	0	JALCNX000000000	SRX12655976	517
UW_MP_ENTER2_10		USB	63	1.0000	115			Illumina NovaSeq S4	91.75	1.34	4.824	127	0.0527	55.7	68	4	0	0	JAKVJS000000000	SRX12656289	393
UW_MP_ENTER2_11		CSTR	12	1.0000	113			Illumina NovaSeq S4	90.54	0.51	4.755	155	0.0372	55.7	66	3	0	0	JALCRS000000000	SRX12654482	422
UW_MP_ENTER3_1	ENTER3	USB	77		125	d__Bacteria;p__Proteobacteria;c__Gammaproteobacteria;o__Enterobacterales;f__Enterobacteriaceae;g__Proteus;s__Proteus vulgaris	GCF_901472505.1	Illumina NovaSeq S4	99.46	0	3.912	30	0.2166	37.8	74	2	0	0	JAKVJC000000000	SRX12656338	913
UW_MP_ENTER4_1	ENTER4	CSTR	108		125	d__Bacteria;p__Proteobacteria;c__Gammaproteobacteria;o__Enterobacterales;f__Enterobacteriaceae;g__Citrobacter;s__Citrobacter braakii	GCF_002075345.1	Illumina NovaSeq S4	98.4	0.71	4.924	34	0.2174	52.1	74	2	0	0	JALCMA000000000	SRX12655904	8,904
UW_MP_ENTER4_2		CSTR	146	1.0000	125			Illumina NovaSeq S4	98.63	0.75	4.96	43	0.18	52.1	74	2	0	0	JALCND000000000	SRX12655903	3,163
UW_MP_ENTER5_1	ENTER5	CSTR	192		117	d__Bacteria;p__Proteobacteria;c__Gammaproteobacteria;o__Enterobacterales;f__Enterobacteriaceae;g__Lelliottia;s__	NA	Illumina NovaSeq S4	94.44	1.07	4.58	116	0.0513	52.8	72	4	0	0	JALCNW000000000	SRX12655976	587
UW_MP_LAC1_1	LAC1	USB	63		119	d__Bacteria;p__Firmicutes;c__Bacilli;o__Lactobacillales;f__Lactobacillaceae;g__Lactobacillus;s__Lactobacillus delbrueckii	GCF_001433875.1	Illumina NovaSeq S4	96.43	0	1.8	62	0.037	50.2	46	4	0	1	JAKVJT000000000	SRX12656289	229
UW_MP_LAC1_2		USB	77	0.9998	116			Illumina NovaSeq S4	92.53	0	1.657	50	0.0451	50.4	51	4	0	1	JAKVJD000000000	SRX12656338	350
UW_MP_LAC1_3		USB	98	0.9997	110			PacBio Sequel II	80.84	0	1.681	2	0.866	49.9	77	7	8	7	JAKVNX000000000	SAMN18243097	1
UW_MP_LAC2_1	LAC2	CSTR	108		113	d__Bacteria;p__Firmicutes;c__Bacilli;o__Lactobacillales;f__Lactobacillaceae;g__Lactobacillus;s__Lactobacillus delbrueckii	GCF_001433875.1	Illumina NovaSeq S4	90.15	0.22	1.512	49	0.0383	50.8	34	1	0	0	JALCLZ000000000	SRX12655904	207
UW_MP_LAC2_2		CSTR	120	0.9999	113			Illumina NovaSeq S4	90.15	0.22	1.62	57	0.0347	50.7	51	2	1	0	JALCMM000000000	SRX12655888	208
UW_MP_LAC2_3		CSTR	146	0.9999	111			Illumina NovaSeq S4	88.2	0.22	1.58	50	0.0389	50.5	55	3	0	0	JALCNC000000000	SRX12655903	413
UW_MP_LAC3_1	LAC3	USB	148		106	d__Bacteria;p__Firmicutes;c__Bacilli;o__Lactobacillales;f__Lactobacillaceae;g__Lactobacillus;s__Lactobacillus delbrueckii	GCF_001433875.1	Illumina NovaSeq S4	83.77	0.09	1.338	41	0.0473	50.7	51	4	0	0	JALCCW000000000	SRX12657082	430
UW_MP_LCO1_1	LCO1	CSTR	44		130	d__Bacteria;p__Firmicutes_A;c__Clostridia;o__Lachnospirales;f__Lachnospiraceae;g__Agathobacter;s__Agathobacter rectalis	GCF_000020605.1	PacBio Sequel II	99.3	0.24	3.246	4	1.6305	41.9	58	5	5	5	JAKVOQ000000000	SAMN18243037	3
UW_MP_LCO1_2		CSTR	36	0.9997	111			Illumina NovaSeq S4	85.99	0	2.152	31	0.1489	41.8	53	0	1	3	JALCSD000000000	SRX12654483	3,051
UW_MP_LCO1_3		CSTR	49	0.9996	110			Illumina NovaSeq S4	87.99	1.21	2.129	92	0.028	42.2	51	0	1	3	JALCSR000000000	SRX12654484	183
UW_MP_LCO2_1	LCO2	CSTR	108		123	d__Bacteria;p__Firmicutes_A;c__Clostridia;o__Lachnospirales;f__Lachnospiraceae;g__Eubacterium_I;s__Eubacterium_I sp000270305	GCF_000270305.1	Illumina NovaSeq S4	97.99	1.15	2.509	32	0.1097	51.1	43	0	0	0	JALCLY000000000	SRX12655904	564
UW_MP_LCO2_2		CSTR	97	1.0000	122			Illumina NovaSeq S4	97.41	1.15	2.476	27	0.1253	51.2	43	0	0	0	JALCLP000000000	SRX12655187	1,796
UW_MP_LCO2_3		CSTR	84	1.0000	115			Illumina NovaSeq S4	89.94	1.15	2.304	22	0.1545	50.9	43	0	0	0	JALCUJ000000000	SRX12655158	1,513
UW_MP_LCO3_1	LCO3	USB	56		116	d__Bacteria;p__Firmicutes_A;c__Clostridia;o__Lachnospirales;f__Lachnospiraceae;g__Butyrivibrio;s__	NA	Illumina NovaSeq S4	94.33	3.01	3.574	60	0.0732	45.1	39	0	0	1	JAKVKH000000000	SRX12656290	427
UW_MP_LCO4_1	LCO4	CSTR	66		114	d__Bacteria;p__Firmicutes_A;c__Clostridia;o__Lachnospirales;f__Lachnospiraceae;g__Eubacterium_H;s__Eubacterium_H sp003488475	GCA_003488475.1	Illumina NovaSeq S4	89.17	0.64	1.907	25	0.1356	50.5	46	0	0	1	JALCTJ000000000	SRX12654503	460
UW_MP_LCO5_1	LCO5	CSTR	0		109	d__Bacteria;p__Firmicutes_A;c__Clostridia;o__Lachnospirales;f__Lachnospiraceae;g__CAG-791;s__	NA	Illumina NovaSeq S4	84.34	0	2.67	27	0.1657	55.0	30	0	0	0	JALCRM000000000	SRX12654481	905
UW_MP_LENLAC1_1	LENLAC1	USB	15		112	d__Bacteria;p__Firmicutes;c__Bacilli;o__Lactobacillales;f__Lactobacillaceae;g__Lentilactobacillus;s__Lentilactobacillus diolivorans	GCF_001434255.1	Illumina NovaSeq S4	92.81	3.07	3.131	149	0.0245	40.0	33	3	1	0	JAKVKX000000000	SRX12655988	389
UW_MP_LEUC1_1	LEUC1	CSTR	60		131	d__Bacteria;p__Firmicutes;c__Bacilli;o__Lactobacillales;f__Lactobacillaceae;g__Leuconostoc;s__Leuconostoc mesenteroides	GCF_000014445.1	PacBio Sequel II	99.74	0	1.856	1	1.856	37.9	71	4	4	4	JAKVOL000000000	SAMN18243139	2
UW_MP_LEUC1_2		CSTR	54	0.9987	123			Illumina NovaSeq S4	98.24	0.53	1.643	39	0.0796	37.8	36	1	0	0	JALCTB000000000	SRX12654502	1,187
UW_MP_LEUC1_3		CSTR	78	0.9933	123			Illumina NovaSeq S4	98.94	0.53	1.863	35	0.0736	37.7	53	0	0	0	JALCTZ000000000	SRX12655143	314
UW_MP_LEUC1_4		CSTR	49	0.9990	115			Illumina NovaSeq S4	89.95	0	1.426	22	0.103	37.9	37	1	0	0	JALCSQ000000000	SRX12654484	988
UW_MP_LEUC1_5		CSTR	72	0.9933	111			Illumina NovaSeq S4	88.29	1.06	1.668	65	0.0316	37.6	35	0	0	0	JALCTQ000000000	SRX12655142	244
UW_MP_LEUC1_6		CSTR	120	0.9970	109			Illumina NovaSeq S4	86.45	1.3	1.496	79	0.0233	37.9	39	0	0	0	JALCML000000000	SRX12655888	134
UW_MP_LEUC1_7		CSTR	44	0.9983	104			PacBio Sequel II	79.39	0	1.571	28	0.0738	37.8	47	1	1	1	JAKVOR000000000	SAMN18243037	1
UW_MP_LIQLAC1_1	LIQLAC1	USB	197		129	d__Bacteria;p__Firmicutes;c__Bacilli;o__Lactobacillales;f__Lactobacillaceae;g__Liquorilactobacillus;s__Liquorilactobacillus nagelii	GCF_001434225.1	PacBio Sequel II	98.95	1.31	2.447	1	2.4466	36.9	57	6	6	6	JALCLK000000000	SRX12729151	4
UW_MP_LIQLAC2_1	LIQLAC2	CSTR	120		119	d__Bacteria;p__Firmicutes;c__Bacilli;o__Lactobacillales;f__Lactobacillaceae;g__Liquorilactobacillus;s__Liquorilactobacillus nagelii	GCF_001434225.1	Illumina NovaSeq S4	95.29	0	2.032	32	0.0932	36.9	51	3	1	0	JALCMK000000000	SRX12655888	1,105
UW_MP_MEG1_1	MEG1	USB	56		115	d__Bacteria;p__Firmicutes_C;c__Negativicutes;o__Veillonellales;f__Megasphaeraceae;g__Megasphaera;s__	NA	Illumina NovaSeq S4	92.22	0.6	1.903	47	0.0574	45.7	43	2	0	0	JAKVKI000000000	SRX12656290	7,120
UW_MP_MEG1_2		USB	63	0.9993	112			Illumina NovaSeq S4	88.62	0.6	1.91	48	0.0736	45.6	45	2	0	0	JAKVJU000000000	SRX12656289	6,875
UW_MP_MEG1_3		USB	21	0.9994	106			Illumina NovaSeq S4	82.53	0	1.754	49	0.0527	45.4	34	3	0	0	JAKVKR000000000	SRX12655989	1,008
UW_MP_MEG2_1	MEG2	CSTR	168		111	d__Bacteria;p__Firmicutes_C;c__Negativicutes;o__Veillonellales;f__Megasphaeraceae;g__Megasphaera_A;s__	GCF_900103535.1	Illumina NovaSeq S4	89.24	1.4	2.097	97	0.0283	39.8	45	1	0	0	JALCNL000000000	SRX12655961	252
UW_MP_MEG2_2		CSTR	146	0.9995	107			Illumina NovaSeq S4	84.43	0.9	1.792	88	0.0272	40.0	38	0	0	0	JALCNB000000000	SRX12655903	144
UW_MP_MEG3_1	MEG3	USB	176		107	d__Bacteria;p__Firmicutes_C;c__Negativicutes;o__Veillonellales;f__Megasphaeraceae;g__Megasphaera;s__	NA	Illumina NovaSeq S4	84.28	0.6	1.687	73	0.0311	46.9	45	1	0	0	JALCDF000000000	SRX12657171	221
UW_MP_MEG3_2		USB	28	0.9989	106			Illumina NovaSeq S4	83.71	0.6	1.669	66	0.0366	46.9	41	1	0	0	JAKVKN000000000	SRX12656283	1,028
UW_MP_MEG4_1	MEG4	USB	28		105	d__Bacteria;p__Firmicutes_C;c__Negativicutes;o__Veillonellales;f__Megasphaeraceae;g__Megasphaera;s__Megasphaera sp000417505	GCF_000417505.1	Illumina NovaSeq S4	84.22	0	1.63	64	0.0352	55.2	50	3	0	0	JAKVKO000000000	SRX12656283	4,503
UW_MP_MEG5_1	MEG5	USB	197		103	d__Bacteria;p__Firmicutes_C;c__Negativicutes;o__Veillonellales;f__Megasphaeraceae;g__Megasphaera;s__Megasphaera cerevisiae	GCF_001045675.1	PacBio Sequel II	76.05	1.87	1.705	7	0.3397	44.5	29	3	3	3	JALCLJ000000000	SRX12729151	17
UW_MP_METH1_1	METH1	CSTR	49		126	d__Archaea;p__Thermoplasmatota;c__Thermoplasmata;o__Methanomassiliicoccales;f__Methanomethylophilaceae;g__Methanomethylophilus;s__Methanomethylophilus sp001481295	GCF_001481295.1	Illumina NovaSeq S4	98.39	0.81	1.744	9	0.3894	60.5	46	2	1	1	JALCSP000000000	SRX12654484	219
UW_MP_METH1_2		CSTR	36	1.0000	124			Illumina NovaSeq S4	97.58	0.81	1.696	9	0.2547	60.5	46	2	1	1	JALCSC000000000	SRX12654483	247
UW_MP_METH1_3		CSTR	44	0.9998	122			PacBio Sequel II	95.97	2.42	1.51	20	0.1119	60.7	41	1	1	1	JAKVOS000000000	SAMN18243037	1
UW_MP_MIC1_1	MIC1	USB	105		105	d__Bacteria;p__Actinobacteriota;c__Actinomycetia;o__Actinomycetales;f__Microbacteriaceae;g__;s__	NA	Illumina NovaSeq S4	83.82	0.58	1.208	53	0.0324	66.2	36	0	0	0	JALCCL000000000	SRX12656339	106
UW_MP_MIC1_2		USB	77	0.9993	103			Illumina NovaSeq S4	81.98	0	1.286	65	0.0223	66.2	38	1	0	1	JAKVJE000000000	SRX12656338	97
UW_MP_MORAX1_1	MORAX1	USB	77		121	d__Bacteria;p__Proteobacteria;c__Gammaproteobacteria;o__Pseudomonadales;f__Moraxellaceae;g__Acinetobacter;s__Acinetobacter populi	GCF_002174125.1	Illumina NovaSeq S4	97.48	0	3.317	62	0.0823	40.3	55	5	0	0	JAKVJF000000000	SRX12656338	361
UW_MP_MORAX2_1	MORAX2	USB	77		109	d__Bacteria;p__Proteobacteria;c__Gammaproteobacteria;o__Pseudomonadales;f__Moraxellaceae;g__Acinetobacter;s__Acinetobacter gerneri	GCF_000368565.1	Illumina NovaSeq S4	84.98	0.86	4.041	48	0.1192	37.6	68	1	0	0	JAKVJG000000000	SRX12656338	670
UW_MP_MUR1_1	MUR1	USB	0		108	d__Bacteria;p__Bacteroidota;c__Bacteroidia;o__Bacteroidales;f__Muribaculaceae;g__;s__	NA	Illumina NovaSeq S4	85.97	0.57	2.948	93	0.0394	44.6	40	0	2	0	JAKVKZ000000000	SRX12655975	301
UW_MP_MYC1_1	MYC1	USB	176		122	d__Bacteria;p__Actinobacteriota;c__Actinomycetia;o__Mycobacteriales;f__Mycobacteriaceae;g__Corynebacterium;s__Corynebacterium provencense	GCF_900049755.1	Illumina NovaSeq S4	96.62	1.91	2.996	29	0.2017	67.1	57	3	0	0	JALCDE000000000	SRX12657171	633
UW_MP_OSCL1_1	OSCL1	CSTR	12		119	d__Bacteria;p__Firmicutes_A;c__Clostridia;o__Oscillospirales;f__Oscillospiraceae;g__Oscillibacter;s__	NA	Illumina NovaSeq S4	96.98	1.34	2.253	69	0.0428	55.4	43	2	0	0	JALCRR000000000	SRX12654482	213
UW_MP_OSCL2_1	OSCL2	CSTR	12		114	d__Bacteria;p__Firmicutes_A;c__Clostridia;o__Oscillospirales;f__Oscillospiraceae;g__Oscillibacter;s__	NA	Illumina NovaSeq S4	93.74	3.02	1.796	63	0.0293	61.7	30	1	0	1	JALCRQ000000000	SRX12654482	183
UW_MP_OSCL3_1	OSCL3	CSTR	36		114	d__Bacteria;p__Firmicutes_A;c__Clostridia;o__Oscillospirales;f__Oscillospiraceae;g__Intestinimonas;s__	NA	Illumina NovaSeq S4	89.09	0.34	2.054	28	0.1131	53.8	38	2	0	0	JALCSB000000000	SRX12654483	1,146
UW_MP_PREV1_1	PREV1	USB	98		128	d__Bacteria;p__Bacteroidota;c__Bacteroidia;o__Bacteroidales;f__Bacteroidaceae;g__Prevotella;s__	NA	PacBio Sequel II	97.64	2.11	3.412	1	3.412	43.8	54	4	4	4	JAKVNY000000000	SAMN18243097	3
UW_MP_PREV2_1	PREV2	USB	42		125	d__Bacteria;p__Bacteroidota;c__Bacteroidia;o__Bacteroidales;f__Bacteroidaceae;g__Prevotella;s__Prevotella sp002409785	GCA_002409785.1	PacBio Sequel II	97.94	1.86	3.131	13	0.3543	44.5	54	4	4	4	JAKVOF000000000	SAMN18243454	7
UW_MP_PREV2_2		CSTR	120	0.9929	121			Illumina NovaSeq S4	97.64	1.6	2.978	54	0.0875	44.2	45	0	1	0	JALCMJ000000000	SRX12655888	699
UW_MP_PREV2_3		CSTR	54	0.9918	121			Illumina NovaSeq S4	97.64	2.11	3.056	49	0.0759	44.2	45	1	0	0	JALCTA000000000	SRX12654502	1,437
UW_MP_PREV2_4		CSTR	49	0.9917	119			Illumina NovaSeq S4	95.61	2.03	3.027	59	0.0706	44.1	39	1	0	0	JALCSO000000000	SRX12654484	455
UW_MP_PREV2_5		CSTR	66	0.9949	117			Illumina NovaSeq S4	94.09	0.68	2.507	63	0.0545	44.9	45	1	0	0	JALCTI000000000	SRX12654503	2,330
UW_MP_PREV2_6		USB	77	0.9980	116			Illumina NovaSeq S4	94.02	1.18	2.298	94	0.0295	45.2	40	0	0	0	JAKVJH000000000	SRX12656338	5,405
UW_MP_PREV2_7		USB	63	0.9980	113			Illumina NovaSeq S4	91.22	1.35	2.266	91	0.0295	45.0	33	0	0	0	JAKVJV000000000	SRX12656289	9,265
UW_MP_PREV2_8		CSTR	192	0.9942	112			Illumina NovaSeq S4	90.26	1.18	2.544	105	0.0299	44.7	43	1	0	0	JALCNV000000000	SRX12655976	1,069
UW_MP_PREV2_9		USB	56	0.9980	111			Illumina NovaSeq S4	89.3	1.69	2.298	90	0.0338	45.3	40	0	0	0	JAKVKJ000000000	SRX12656290	4,804
UW_MP_PREV2_10		CSTR	168	0.9935	111			Illumina NovaSeq S4	89.5	1.53	2.566	114	0.0271	44.4	38	1	0	0	JALCNK000000000	SRX12655961	275
UW_MP_PREV2_11		CSTR	72	0.9943	111			Illumina NovaSeq S4	87.92	0.34	2.374	93	0.0358	44.9	43	1	0	0	JALCTP000000000	SRX12655142	1,346
UW_MP_PREV2_12		CSTR	126	0.9939	110			Illumina NovaSeq S4	86.71	1.1	2.521	63	0.0507	44.4	37	0	0	0	JALCMT000000000	SRX12655887	333
UW_MP_PREV2_13		CSTR	84	0.9952	109			Illumina NovaSeq S4	87.09	1.76	2.21	114	0.0242	45.0	37	0	0	0	JALCUI000000000	SRX12655158	1,338
UW_MP_PREV2_14		CSTR	78	0.9964	108			Illumina NovaSeq S4	85.57	0.68	2.115	92	0.0287	45.2	41	0	0	0	JALCTY000000000	SRX12655143	1,522
UW_MP_PREV2_15		CSTR	146	0.9930	106			Illumina NovaSeq S4	84.82	2.14	2.501	128	0.0234	44.4	41	0	0	0	JALCNA000000000	SRX12655903	539
UW_MP_PREV3_1	PREV3	CSTR	44		124	d__Bacteria;p__Bacteroidota;c__Bacteroidia;o__Bacteroidales;f__Bacteroidaceae;g__Prevotella;s__Prevotella sp900316015	GCA_900316015.1	PacBio Sequel II	94.29	1.07	3.459	6	0.9289	46.9	48	4	4	4	JAKVOT000000000	SAMN18243037	2
UW_MP_PREV3_2		CSTR	49	0.9997	117			Illumina NovaSeq S4	95	0.71	2.917	85	0.0392	47.0	41	0	0	0	JALCSN000000000	SRX12654484	792
UW_MP_PREV3_3		CSTR	36	0.9996	117			Illumina NovaSeq S4	94.29	0.71	2.84	83	0.0393	47.0	37	0	0	0	JALCSA000000000	SRX12654483	611
UW_MP_PREV4_1	PREV4	USB	63		113	d__Bacteria;p__Bacteroidota;c__Bacteroidia;o__Bacteroidales;f__Bacteroidaceae;g__Prevotella;s__	NA	Illumina NovaSeq S4	89.67	1.01	2.933	73	0.0684	40.2	45	1	1	1	JAKVJW000000000	SRX12656289	514
UW_MP_PREV4_2		USB	56	0.9995	113			Illumina NovaSeq S4	89.86	2.36	2.945	99	0.0482	40.2	48	2	1	0	JAKVKK000000000	SRX12656290	280
UW_MP_PREV4_3		USB	77	0.9991	110			Illumina NovaSeq S4	84.97	1.77	2.998	69	0.0938	40.2	45	0	1	0	JAKVJI000000000	SRX12656338	714
UW_MP_PREV5_1	PREV5	USB	0		110	d__Bacteria;p__Bacteroidota;c__Bacteroidia;o__Bacteroidales;f__Bacteroidaceae;g__Prevotella;s__	NA	Illumina NovaSeq S4	84.68	0.66	2.611	37	0.1869	43.7	40	2	0	0	JAKVLA000000000	SRX12655975	1,748
UW_MP_PREV6_1	PREV6	USB	98		107	d__Bacteria;p__Bacteroidota;c__Bacteroidia;o__Bacteroidales;f__Bacteroidaceae;g__Prevotella;s__Prevotella sp002409785	GCA_002409785.1	PacBio Sequel II	82.21	1.71	2.42	24	0.1166	44.7	48	3	3	3	JAKVNZ000000000	SAMN18243097	2
UW_MP_PREV7_1	PREV7	USB	197		106	d__Bacteria;p__Bacteroidota;c__Bacteroidia;o__Bacteroidales;f__Bacteroidaceae;g__Prevotella;s__	NA	PacBio Sequel II	77.7	4.48	2.524	15	0.32	40.5	51	4	4	4	JALCLI000000000	SRX12729151	17
UW_MP_PREV8_1	PREV8	USB	176		101	d__Bacteria;p__Bacteroidota;c__Bacteroidia;o__Bacteroidales;f__Bacteroidaceae;g__Prevotella;s__	NA	Illumina NovaSeq S4	80.41	2.45	1.952	82	0.0302	44.8	43	0	0	0	JALCDD000000000	SRX12657171	1,995
UW_MP_PROP1_1	PROP1	USB	197		117	d__Bacteria;p__Actinobacteriota;c__Actinomycetia;o__Propionibacteriales;f__Propionibacteriaceae;g__Acidipropionibacterium;s__	NA	PacBio Sequel II	91.48	1.32	3.682	26	0.2395	67.8	55	1	1	1	JALCLH000000000	SRX12729151	7
UW_MP_RUM1_1	RUM1	CSTR	12		118	d__Bacteria;p__Firmicutes_A;c__Clostridia;o__Oscillospirales;f__Ruminococcaceae;g__Faecalibacterium;s__	NA	Illumina NovaSeq S4	93.88	0	2.218	35	0.1048	56.5	46	1	1	0	JALCRP000000000	SRX12654482	535
UW_MP_SACCH1_1	SACCH1	USB	0		126	d__Bacteria;p__Firmicutes_A;c__Clostridia;o__Saccharofermentanales;f__Saccharofermentanaceae;g__Saccharofermentans;s__	NA	Illumina NovaSeq S4	99.65	0	1.923	12	0.2265	46.7	42	2	0	1	JAKVLB000000000	SRX12655975	432
UW_MP_SACCH1_2		CSTR	0	0.9997	111			Illumina NovaSeq S4	86.88	0	1.394	21	0.078	46.2	37	1	1	0	JALCRL000000000	SRX12654481	269
UW_MP_SCHLAC1_1	SCHLAC1	CSTR	192		126	d__Bacteria;p__Firmicutes;c__Bacilli;o__Lactobacillales;f__Lactobacillaceae;g__Schleiferilactobacillus;s__Schleiferilactobacillus harbinensis	GCF_000425885.1	Illumina NovaSeq S4	98.69	1.05	3.131	16	0.3078	53.5	60	3	0	0	JALCNU000000000	SRX12655976	579
UW_MP_SCHLAC1_2		CSTR	126	0.9998	110			Illumina NovaSeq S4	85.69	1.05	2.478	55	0.0637	53.8	42	1	1	0	JALCMS000000000	SRX12655887	379
UW_MP_SCHLAC1_3		CSTR	120	0.9995	109			Illumina NovaSeq S4	85.42	0.52	2.468	79	0.0426	53.7	26	1	0	0	JALCMI000000000	SRX12655888	243
UW_MP_SCHLAC2_1	SCHLAC2	CSTR	78		119	d__Bacteria;p__Firmicutes;c__Bacilli;o__Lactobacillales;f__Lactobacillaceae;g__Schleiferilactobacillus;s__Schleiferilactobacillus perolens	GCF_001435585.1	Illumina NovaSeq S4	95.29	0.79	2.837	42	0.108	49.4	25	2	0	0	JALCTX000000000	SRX12655143	290
UW_MP_SELEN1_1	SELEN1	USB	42		115	d__Bacteria;p__Firmicutes_C;c__Negativicutes;o__Selenomonadales;f__Selenomonadaceae;g__Selenomonas_A;s__	NA	PacBio Sequel II	90.69	1.87	2.941	22	0.2326	51.7	71	8	6	8	JAKVOG000000000	SAMN18243454	6
UW_MP_SPH1_1	SPH1	CSTR	44		130	d__Bacteria;p__Spirochaetota;c__Spirochaetia;o__Sphaerochaetales;f__Sphaerochaetaceae;g__RZYO01;s__	NA	PacBio Sequel II	98.85	0	3.468	3	3.4088	44.4	55	6	5	5	JAKVOU000000000	SAMN18243037	4
UW_MP_SPH2_1	SPH2	CSTR	44		125	d__Bacteria;p__Spirochaetota;c__Spirochaetia;o__Sphaerochaetales;f__Sphaerochaetaceae;g__RUG023;s__	NA	PacBio Sequel II	97.63	2.3	2.472	20	0.1891	55.9	44	2	2	1	JAKVOV000000000	SAMN18243037	17
UW_MP_SPH2_2		CSTR	24	0.9952	123			PacBio Sequel II	96.48	1.15	2.251	14	0.2435	56.2	46	4	3	3	JAKVPA000000000	SAMN18243588	11
UW_MP_SPH2_3		CSTR	54	0.9962	116			Illumina NovaSeq S4	94.19	0	1.763	75	0.0264	56.9	31	2	0	0	JALCSZ000000000	SRX12654502	2,865
UW_MP_SPH2_4		CSTR	49	0.9958	111			Illumina NovaSeq S4	88.44	0	1.721	64	0.0348	57.0	26	2	0	0	JALCSM000000000	SRX12654484	5,420
UW_MP_SPH2_5		CSTR	36	0.9956	106			Illumina NovaSeq S4	83.84	0	1.538	64	0.0255	56.9	27	2	0	0	JALCRZ000000000	SRX12654483	1,0252
UW_MP_SPH2_6		CSTR	12	0.9976	100			Illumina NovaSeq S4	78.09	0	1.336	76	0.0202	56.9	27	0	0	0	JALCRO000000000	SRX12654482	1,119
UW_MP_SPH2_7		CSTR	66	0.9947	98			Illumina NovaSeq S4	76.37	0	1.635	91	0.0202	56.7	34	2	1	1	JALCTH000000000	SRX12654503	343
UW_MP_SPHING1_1	SPHING1	USB	0		122	d__Bacteria;p__Proteobacteria;c__Alphaproteobacteria;o__Sphingomonadales;f__Sphingomonadaceae;g__Sphingobium;s__	NA	Illumina NovaSeq S4	98.11	0.78	2.808	48	0.0789	63.3	46	1	1	1	JAKVLC000000000	SRX12655975	488
UW_MP_SPHING1_2		CSTR	0	0.9999	121			Illumina NovaSeq S4	97.69	1.64	3.059	62	0.0623	63.2	49	1	2	2	JALCRK000000000	SRX12654481	405
UW_MP_SPOR1_1	SPOR1	CSTR	192		120	d__Bacteria;p__Firmicutes;c__Bacilli;o__Bacillales_G;f__Sporolactobacillaceae;g__Sporolactobacillus;s__Sporolactobacillus sp900543345	GCA_900543345.1	Illumina NovaSeq S4	96.74	1.94	3.636	39	0.1143	49.1	41	5	2	0	JALCNT000000000	SRX12655976	510
UW_MP_STREP1_1	STREP1	CSTR	78		122	d__Bacteria;p__Firmicutes;c__Bacilli;o__Lactobacillales;f__Streptococcaceae;g__Lactococcus;s__Lactococcus lactis	GCF_900099625.1	Illumina NovaSeq S4	98.71	0.57	2.274	67	0.0391	35.0	51	3	0	0	JALCTW000000000	SRX12655143	206
UW_MP_STREP1_2		CSTR	49	0.9999	120			Illumina NovaSeq S4	97.16	0.57	2.402	84	0.0436	34.9	39	1	0	0	JALCSL000000000	SRX12654484	220
UW_MP_STREP1_3		CSTR	72	0.9997	109			Illumina NovaSeq S4	87.08	0.82	2.106	95	0.0273	35.0	43	2	0	0	JALCTO000000000	SRX12655142	216
UW_MP_STREP2_1	STREP2	USB	28		118	d__Bacteria;p__Firmicutes;c__Bacilli;o__Lactobacillales;f__Streptococcaceae;g__MMGLQ5-1;s__	NA	Illumina NovaSeq S4	94.04	0.66	2.517	41	0.0783	35.1	42	1	0	0	JAKVKP000000000	SRX12656283	238
UW_MP_STREP2_2		USB	21	0.9999	104			Illumina NovaSeq S4	78.81	0.66	2.101	30	0.1097	35.2	4	1	0	0	JAKVKS000000000	SRX12655989	491
UW_MP_STREP3_1	STREP3	CSTR	192		115	d__Bacteria;p__Firmicutes;c__Bacilli;o__Lactobacillales;f__Streptococcaceae;g__Lactococcus;s__Lactococcus lactis_E	GCF_002078765.2	Illumina NovaSeq S4	91.32	0	2.102	39	0.0881	35.7	43	2	0	0	JALCNS000000000	SRX12655976	250
UW_MP_STREP4_1	STREP4	USB	21		114	d__Bacteria;p__Firmicutes;c__Bacilli;o__Lactobacillales;f__Streptococcaceae;g__Lactococcus_A;s__Lactococcus_A raffinolactis	GCF_001591765.1	Illumina NovaSeq S4	90.57	0.5	1.911	37	0.0827	40.4	29	1	0	0	JAKVKT000000000	SRX12655989	579
UW_MP_TREP1_1	TREP1	USB	105		107	d__Bacteria;p__Spirochaetota;c__Spirochaetia;o__Treponematales;f__Treponemataceae;g__Treponema_D;s__	NA	Illumina NovaSeq S4	84.62	0.35	2.692	95	0.0361	44.8	43	0	0	0	JALCCK000000000	SRX12656339	367

a Strain name assigned to each MAG. The UW_MP prefix stands for University of Wisconsin Milk Permeate bioreactor. MAGs are clustered during dereplication using dRep (11). Strains with a numerical suffix of _1 are the highest-quality dRep representative MAGs for a given cluster; nonrepresentative MAGs in each cluster are assigned the same strain name with sequential numerical suffixes (e.g., _2 and _3), assigned in order of decreasing quality, according to the dRep score.

b ACET, *Acetobacter*; ACID, *Acidaminococcaceae*; ACT, *Actinomycetaceae*; ACUT, *Acutalibacteraceae*; AGRLAC, *Agrilactobacillus*; ANA, *Anaerovoracaceae*; ATO, *Atopobiaceae*; BACIL, *Bacilli*; BACTE, *Bacteroidales*; BIF, *Bifidobacterium*; BUL, *Bulleidia*; BURK, *Burkholderiaceae*; CARN, *Carnobacteriaceae*; CAUL, *Caulobacteraceae*; CLOS, *Clostridium*; EGG, *Eggerthellaceae*; ENTER, Enterobacteriaceae; LAC, *Lactobacillus*; LCO, *Lachnospiraceae*; LENLAC, *Lentilactobacillus*; LEUC, *Lecuonostoc*; LIQLAC, *Liquorilactobacillus*; MEG, *Megasphaera*; METH, *Methanomethylophilus*; MIC, *Microbacteriaceae*; MORAX, *Moraxellaceae*; MUR, *Muribaculaceae*; MYC, *Mycobacteriaceae*; OSCL, *Oscillospiraceae*; PREV, *Prevotella*; PROP, *Propionibacteriaceae*; RUM, *Ruminococcaceae*; SACCH, *Saccharofermentans*; SCHLAC, *Schleiferilactobacillus*; SELEN, *Selenomonadaceae*; SPH, *Sphaerochaetaceae*; SPHING, *Sphingobium*; SPOR, *Sporolactobacillaceae*; STREP, *Streptococcaceae*; TREP, Treponema.

c Sample from which a given MAG was derived, described as the bioreactor of origin and the sampling day, where day 0 is designated as the day the bioreactor was inoculated. Each bioreactor was fed ultrafiltered milk permeate amended with 400 mg/L of N, in the form of NH_4_Cl. The CSTR was operated at pH 5.5 and 35°C, with a 6-day solids/hydraulic retention time. The USB was operated at pH 5.5 and 21°C, with a 1.4-day hydraulic retention time and with dynamic solids removal.

d Average nucleotide identity (ANI) between representative MAG and other MAGs included in the same cluster by dRep.

e dRep scoring calculation: (*A* × completeness) − (*B* × contamination) + [*C* × [contamination × (strain heterogeneity/100)]] + [*D* × log(*N*_50_)] + [*E* × log(genome size)] + [*F* × (centrality – ANI)], where *A* to *F* were weighted with values of 1, 0.5, 1, 5, 0, and 1, respectively.

f NCBI GenBank accession number of the reference genome in GTDB-Tk (13) that is closest to the representative MAG. NA, not applicable, i.e., MAGs without a closely matched reference genome when using the default minimum alignment fraction of 0.65.

g NCBI GenBank accession number for each reported MAG.

h NCBI SRA accession number for the raw reads of the metagenome for each reported MAG. For PacBio samples that utilized multiple runs, the NCBI BioSample for the experiment is provided.

i The number of raw reads that aligned to each reported MAG with BBMap or minimap2 for Illumina and PacBio reads, respectively. These reads originated from the metagenome in which the MAG was assembled.

### Data availability.

Raw metagenomic sequence data and MAGs for each sample are available at NCBI GenBank under BioProject accession number PRJNA768492. NCBI genome accession numbers for all 278 MAGs are displayed in Table 1. All information on library construction and sequencing can be found at https://img.jgi.doe.gov using JGI GOLD Study identification number Gs0150020. All custom scripts are available on GitHub (https://github.com/GLBRC/metagenome_analysis).
